# Metabolic Changes in Occipital Lobe Epilepsy with Automatisms

**DOI:** 10.3389/fneur.2014.00135

**Published:** 2014-07-22

**Authors:** Chong H. Wong, Armin Mohamed, Lingfeng Wen, Stefan Eberl, Ernest Somerville, Michael Fulham, Andrew F. Bleasel

**Affiliations:** ^1^Sydney Medical School, University of Sydney, Sydney, NSW, Australia; ^2^Department of Neurology, Westmead Hospital, Westmead, NSW, Australia; ^3^Department of PET and Nuclear Medicine, Royal Prince Alfred Hospital, Camperdown, NSW, Australia; ^4^School of Information Technologies, University of Sydney, Sydney, NSW, Australia; ^5^Institute of Neurological Sciences, Prince of Wales Hospital, Randwick, NSW, Australia

**Keywords:** epilepsies, focal, epilepsy, occipital lobe, positron-emission tomography, fluorodeoxyglucose, automatisms, automotor seizure

## Abstract

**Purpose:** Some studies suggest that the pattern of glucose hypometabolism relates not only to the ictal-onset zone but also reflects seizure propagation. We investigated metabolic changes in patients with occipital lobe epilepsy (OLE) that may reflect propagation of ictal discharge during seizures with automatisms.

**Methods:** Fifteen patients who had undergone epilepsy surgery for intractable OLE and had undergone interictal Fluorine-18-fluorodeoxyglucose positron-emission tomography (^18^F-FDG-PET) between 1994 and 2004 were divided into two groups (with and without automatisms during seizure). Significant regions of hypometabolism were identified by comparing ^18^F-FDG-PET results from each group with 16 healthy controls by using statistical parametric mapping.

**Key Findings:** Significant hypometabolism was confined largely to the epileptogenic occipital lobe in the patient group without automatisms. In patients with automatisms, glucose hypometabolism extended from the epileptogenic occipital lobe into the ipsilateral temporal lobe.

**Significance:** We identified a distinctive hypometabolic pattern that was specific for OLE patients with automatisms during a seizure. This finding supports the postulate that seizure propagation is a cause of glucose hypometabolism beyond the region of seizure onset.

## Introduction

Fluorine-18-fluorodeoxyglucose positron-emission tomography (^18^F-FDG-PET) is often used for localization of the epileptogenic region during an epilepsy presurgical evaluation. However, the interictal ^18^F-FDG-PET hypometabolism often extends beyond the epileptogenic region into the adjacent and remote cortical and subcortical structures (1). Several studies investigating this phenomenon demonstrated the patterns of interictal glucose hypometabolism may reflect metabolic disturbances from propagation of seizures to functionally connected remote brain areas or networks (2–6).

In occipital lobe epilepsy (OLE), oral and manual automatisms are common and are often attributed to seizure propagation from the occipital to the temporal lobe (7, 8). This study examines ^18^F-FDG-PET of patients with OLE to identify specific pattern of functional disturbances in brain areas that may reflect an ictal pathway during seizures with automatisms.

## Materials and Methods

We included all 15 patients with medically intractable epilepsy, who had undergone occipital lobe resections and interictal ^18^F-FDG-PET between 1994 and 2004 at the Westmead and Royal Prince Alfred Hospitals, Australia. The study was approved by the ethics committees in Central Sydney (Protocol no. X03-0161) and Western Sydney Area Health Services [HREC reference no: HS/TG HREC 2003/6/4.13 (1670)]. Patients with resection of the parieto-occipital cortex were not included. The occipital lobe boundaries were established as defined by the Tzourio-Mazoyer atlas (9).

Seizure semiology was determined from the review of the in-patient video-EEG recording and was classified according to the seizure semiologic classification developed at the Cleveland Clinic Foundation in the 1990s (10). Based on the presence or absence of seizures with automatisms during in-patient video-EEG recording, patients were assigned into a seizure with automatisms group or a seizure without automatisms group. Seizures with automatisms were defined as seizures with oro-alimentary and manual automatisms, usually, but not always, with impairment of consciousness (10, 11).

Methods for FDG-PET acquisition and for statistical parametric mapping (SPM2; Wellcome Department of Cognitive Neurology, UK) and spatial pre-processing of FDG-PET images of patients and 16 healthy controls have been described in detail elsewhere (6, 12, 13). In brief, ^18^F-FDG-PET images were realigned, spatially normalized, and smoothed by convolution with a 10-mm FWHM Gaussian kernel. The ^18^F-FDG-PET images of patients with left occipital ictal onset were transposed horizontally so that all ictal-onset foci were lateralized to the right side. Both patients and controls had regional metabolic rates of glucose hypometabolism estimated using a population-based input function calibrated by using two arterialized-venous blood sampling procedures (14). For statistical analysis, ^18^F-FDG-PET images of each patient group were compared with images of the 16 healthy controls (8 males, median age 31.5 years, interquartile range 25–75%; 25–42 years) at the voxel-by-voxel level using two-sample *t*-tests to identify all clusters of voxels exhibiting significant hypometabolism. SPM analysis identifies regions with cluster size (extent threshold) larger than 250 contiguous voxels and with voxel-level significance (height threshold) of *p* ≥ 0.01. Only regions with clusters of voxels that exceeds this extent and height threshold and achieved corrected cluster-level significance of *p* < 0.05 were considered significant. Group analysis was performed to eliminate interindividual metabolic variability. The analysis allowed the identification and comparison of hypometabolic patterns for the seizures with automatisms and the seizures without automatisms groups when compared to normal healthy controls.

## Results

Fifteen patients (eight females and seven males) with age of seizure onset between 2 and 20 years (median age 11 years; IQR 25–75%, 6–17 years) were studied. The median age of the study population was 25 years old (IQR 25–75%, 15–27 years). Ten patients were admitted to hospital for in-patient prolonged video EEG on at least two occasions (range 1–4 video-EEG monitoring). The median number of seizures recorded was 16 seizures (IQR 25–75%, 11–35 seizures; range 10–50 seizures). Among the patients, 13 had intracranial video-EEG studies and showed an ictal-onset zone within the occipital lobe. Two patients (patient 6 and 13) did not undergo invasive monitoring but showed a MRI abnormality with concordant scalp video-EEG findings; both became seizure free after surgery. The median follow-up duration following surgery was 10.1 years (IQR 25–75%, 8–15.8 years). Twelve patients achieved Engel class 1 outcome, 2 had significant seizure improvement (Engel 2) and 1 had worthwhile improvement (Engel 3). The histopathology was summarized in Table 1.

**Table 1:** **Summary of clinical features, investigation results, and surgical outcome**.

Patient (age/gender)	Age of onset	MRI findings	Lobe of ictal onset^a^	Typical semiology^b^	Seizure outcome	Pathology
**PATIENTS WITHOUT AUTOMATISM DURING SEIZURES**						
1. 25 yr/M	12yr	Lt mesial occipital lesion	Lt OLE	Cephalic aura > tonic seizure (Rt arm)	Engel 2	Type 1 cortical dysplasia
2. 20 yr/F	7 yr	Rt inf-mesial occipital lobe lesion	Rt OLE	Visual aura > Lt versive seizure > dialeptic seizure	Engel 1	Type 2 cortical dysplasia
3. 15 yr/M	12 yr	Normal MRI	Rt OLE	Visual aura > Lt versive seizure > SGTCS	Engel 1	Type 2 cortical dysplasia
4. 32 yr/M	18 yr	Rt inf-mesial occipital lobe lesion	Rt OLE	Visual aura > SGTCS	Engel 2	Gliosis
5. 30 yr/F	20 yr	Normal MRI	Lt OLE	Visual aura > SGTCS	Engel 1	Type 1 cortical dysplasia
6. 25 yr/F	17 yr	Lt dorsolateral occipital lobe lesion	Lt OLE	SGTCS	Engel 1	Ganglioglioma
**PATIENTS WITH AUTOMATISMS DURING SEIZURES**						
7. 15 yr/F	4 yr	Normal MRI	Rt OLE	Visual aura > automotor seizure	Engel 1	Type 2 cortical dysplasia
8. 10 yr/F	6 yr	Rt inf-mesial occipital lobe atrophy	Rt OLE	Visual aura > automotor seizure	Engel 1	Gliosis
9. 15 yr/F	3 yr	Rt inf-mesial occipital lobe lesion	Rt OLE	Visual aura > automotor seizure	Engel 1	Ganglioglioma
10. 18 yr/M	6 yr	Normal MRI	Lt OLE	Visual aura > automotor seizure	Engel 1	Type 2 cortical dysplasia
11. 26 yr/M	7 yr	Lt inf-mesial occipital lobe lesion	Lt OLE	Visual aura > automotor seizure > tonic seizure (Rt arm)	Engel 1	Dysembryoplastic neuroepithelial tumor
12. 26 yr/F	11 yr	Normal MRI	Lt OLE	Cephalic aura > automotor seizures > SGTCS	Engel 1	Type 1 cortical dysplasia
13. 13 yr/F	2 yr	Lt dorsolateral occipital lobe lesion	Rt OLE	Visual aura > automotor seizure	Engel 1	Dysembryoplastic neuroepithelial tumor
14. 30 yr/M	17 yr	Rt inf-mesial occipital lobe lesion	Rt OLE	Visual aura > automotor seizure	Engel 3	Ganglioglioma
15. 27 yr/M	14 yr	Normal MRI	Rt OLE	Visual aura > automotor seizure	Engel 2	Type 1 cortical dysplasia

*^a^Origin of seizures was based on clinical history, scalp, and intracranial video-EEG monitoring, MRI, ^18^F-FDG-PET, ^99m^Tc-hexamethyl-propylene-amine-oxime single photon emission computed tomography and neuropsychological studies*.

*^b^Seizure semiology determined on video review of in-patient video-electroencephalography and classified according to the seizure semiologic classification (10). Automotor seizures refer to seizures with oro-alimentary and manual automatisms*.

*Cephalic aura refers a sensation in the head (15)*.

*M, male; F, female; Lt, left; Rt, right; yr, years; inf, inferior; OLE, occipital lobe epilepsy; SGTCS, secondarily generalized tonic clonic seizures*.

Nine patients had seizures with automatisms occurring as a component of their habitual seizures. The other six patients, who never had automatisms as a feature of their habitual seizures, were assigned to the group without automatisms for SPM group analysis. Table 1 summarizes the seizure semiology of all patients studied.

**Figure 1:**
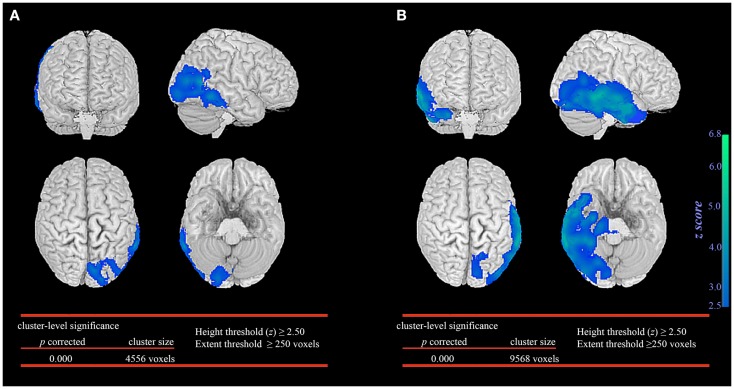
**Statistical parametric mapping comparison between patient groups and healthy controls**. Significant glucose hypometabolism was observed in the ipsilateral occipital lobe in the group of occipital lobe epilepsy patients without automatisms during seizures **(A)**. In the group of occipital lobe epilepsy patients with automatisms during seizures, significant decrease in glucose hypometabolism extends to involve the ispsilateral temporal lobe **(B)**.

In the seizure without automatisms group, SPM analysis revealed significant glucose hypometabolism involving primarily the epileptogenic occipital lobe and extending marginally into the posterior temporal region (Figure 1A). In contrast, the patient group with automatisms not only demonstrated prominent glucose hypometabolism in the epileptogenic occipital lobe but also a significant decrease in glucose metabolism in the basal temporal, lateral temporal, and anteromesial temporal structures (Figure 1B).

The extent of temporal lobe involvement was significantly associated with the presence of automatisms during seizures (*p* < 0.001, median 1223 voxels; IQR 928–4207 voxels) when compared to patients without automatisms during seizures (median 101 voxels; IQR 0–300 voxels). No significant association was found between the extent of temporal lobe involvement, duration of epilepsy before FDG-PET, age when FDG-PET was performed and seizure outcome.

## Discussion

In this study, we determined the interictal metabolic patterns of glucose in patients with OLE with and without automatisms. The major difference in OLE patients with automatisms was the presence of significant glucose hypometabolism in the temporal lobe. We suggest that this interictal metabolic change reflects evidence for the propagation pathway of seizures in patients with automatisms.

Significant hypometabolism was present in the occipital lobe in both groups of patients with OLE. ^18^F-FDG-PET has been used to localize ictal focus by showing regional glucose hypometabolism in the epileptogenic occipital lobe in OLE (16, 17). Our finding was not unexpected given that ^18^F-FDG-PET revealed focal areas of relative hypometabolism that was associated with the epileptogenic zone.

The occipital lobe is connected to the mesial and lateral temporal structures by abundant multisynaptic projections (18, 19). Several studies have shown seizures originating from the occipital lobe readily propagate to the temporal lobe (7, 8, 20, 21), and the occipital to temporal seizure spread coincides with the appearance of oral and manual automatisms (7, 8, 22). Our patient group with seizures and automatisms demonstrated significant glucose hypometabolism extending from the epileptogenic occipital lobe into the temporal lobe. We postulate this hypometabolism reflects neuronal dysfunction from the spread of electrical activity into the ipsilateral temporal lobe during the evolution of seizures with automatisms.

Several studies support the hypothesis that the topography of glucose hypometabolism relates, at least in part, to brain regions involved in the ictal onset and to pathways of seizure propagation generating the clinical manifestations (3, 4, 23). Schlaug et al. examined the relationship between seizure semiology and interictal abnormalities in cerebral glucose metabolism in 48 patients with neocortical focal epilepsy. The investigators found patients with focal clonic seizures had prominent glucose hypometabolism in the contralateral primary motor area and unilateral tonic seizures were associated with markedly decreased metabolism in the supplementary motor area (2). Others reported ictal dystonic posturing to be correlated with contralateral basal ganglia hypometabolism (3, 5). Several brain regions have been reported to produce automatisms by direct cortical electrical stimulation. These include the amygdala, hippocampus, peri-insular temporal cortex, anterior cingulate gyrus, and mesial frontal cortex (24–26). In our cohort of OLE patients with oral and manual automatisms, our analysis found extension of interictal glucose hypometabolism outside of the epileptogenic occipital lobe into basal, lateral, and anteromesial temporal cortices. These structures overlap with regions described in the literature as regions producing automatisms with electrical stimulation. These findings provide confirmatory evidence of occipital lobe seizures often spread to the temporal lobe, and oral and manual automatisms can be a marker of the spread. These same patients overall had a good outcome following surgery on the occipital lobe, leaving the temporal lobe *in situ*. This suggests involvement of the temporal lobe reflects spread of ictal activity, rather than the temporal lobe being a key part of the epileptogenic network.

## Conflict of Interest Statement

The authors declare that the research was conducted in the absence of any commercial or financial relationships that could be construed as a potential conflict of interest.
